# Composite nanoparticle-based vesicles achieve enhanced delivery effects of the natural plant extract of the root, stem, and fruit

**DOI:** 10.3389/fchem.2025.1552298

**Published:** 2025-03-17

**Authors:** Xiaodong Zhuang, Ting Ma, Risheng Liu, Xingyue Fang, Liangjiu Huang

**Affiliations:** ^1^ Department of Clinical Pharmacy, Hainan Cancer Hospital, Haikou, China; ^2^ Department of Pharmacy and Engineering Research Center of Tropical Medicine Innovation and Transformation, The First Affiliated Hospital of Hainan Medical University, Hainan Medical University, Haikou, China

**Keywords:** ginseng, *Millettia speciosa* Champ., *Alpinia oxyphylla* Miq., calcium carbonate nanoparticles, drug delivery

## Abstract

The extract of medicinal plants is increasingly popular around the whole world due to its attractive therapeutic effects. However, the bioavailability of the extract of bioactive compounds was barely satisfactory due to its easily deactivated and untargeted properties. The use of nanotechnology to develop novel carrier delivery techniques for bioactive extracts has been proven to have significant potential and provides an amazing improvement in the therapeutic effect. Calcium carbonate nanoparticles (CaCO_3_ NPs), as representative biodegradable materials, are well recognized as environmentally responsive delivery vehicles for disease treatment. In this study, extracts of the root of ginseng, the fruit of *Alpinia oxyphylla* Miq., and the stem of *Millettia speciosa* Champ. were developed as a CaCO_3_ nanoparticle loading drug. All of the three composite nanoparticles exhibited spherical shapes with a narrow size distribution. Notably, the ginseng extract-loaded CaCO_3_ NPs hold a relatively higher entrapment efficiency of up to 55.2% ± 6.7% and excellent release performance under acidic conditions (pH = 5.5). Moreover, intravenous injection of ginseng CaCO_3_ NPs resulted in significantly enhanced therapeutic effects in the treatment of glioma. The results demonstrate that CaCO_3_-based composite nanoparticles are ideal for the delivery of plant extracts, and the systems are expected to be effective against various types of diseases in the future.

## 1 Introduction

Throughout history, medicinal plants have demonstrated efficacy in augmenting immune function and cognitive abilities when used appropriately. Bioactive compounds such as polysaccharides, saponins, flavonoids, polyphenols, and volatile oils found in medicinal plant extracts significantly contribute to their health-promoting properties (Harvey et al., 2015). With continuous research and development of techniques, medicinal plant extracts have become increasingly prominent in the global market as plant-based herbal remedies for a range of therapeutic applications owing to their anticancer, anti-aging, anti-inflammatory, and hepatoprotective properties and play a role in immune response, cognition, and neurological disorders (Zhang et al., 2024; Zhao et al., 2021). Most traditional herbal medicines contain crude extracts that have multiple potencies in treating various diseases. Within the extensive array of traditional medicinal plants, several *Millettia* species within the Leguminosae family have been investigated to identify bioactive constituents, including phenylpropanoids, terpenoids, alkaloids, flavonoids, and chalcones (Huang et al., 2022; Chen et al., 2016). *Millettia speciosa* Champ. stands out as one of the most extensively studied species, with its roots serving as a folk medicine of considerable economic and medicinal significance. This species is acknowledged for its dual-purpose application as both a culinary herb and a medicinal plant, and it is predominantly found in tropical and subtropical regions, particularly in southeastern China. More than 50 active compounds have been isolated successfully to verify the immunomodulatory, antioxidant, analgesic, and anti-hepatitis activities of the aqueous extracts of the stem of *M. speciosa* Champ. (Xie et al., 2024; Zhang et al., 2021; Lai et al., 2023). In clinical settings, *M. speciosa* Champ. is predominantly used for the treatment of various disorders, including lumbar muscle strain, cough associated with lung deficiency, rheumatoid arthritis, traumatic injuries, and other pathologies (Chen J. et al., 2021; Zhao et al., 2017). Recently, active compounds derived from *M. speciosa* Champ. were applied in cancer therapy and have shown excellent therapeutic effects (Xie et al., 2024; Chen J. et al., 2021). Another medically valuable understory plant in southern China is *Alpinia oxyphylla* Miq., which is expected to exert broadly therapeutic effects in disease treatment. The fruits of the *A. oxyphylla* are recognized as one of “the four famous south medicines” and are used in neuroprotective, anti-inflammatory, anti-nociceptive, and anti-diuretic applications (Yu et al., 2003; Zhang et al., 2018). Among plant species whose roots are employed for medicinal purposes, ginseng is one of the most popular and acclaimed herbs around the world (Chang-Xiao and Pei-Gen, 1992; Wong et al., 2015). Due to its medicinal properties, which include anticancer, antidiabetic, and immunomodulatory effects, as well as its contribution to stress relief (Kim et al., 2018; Huang et al., 2023; Balusamy et al., 2023), ginseng is extensively utilized as dietary supplements, personal care products, pharmaceuticals, and oral care products. Recent clinical investigations have demonstrated the prophylactic and therapeutic potential of ginseng against cancer. Numerous *in vitro* studies have elucidated the high anticancerous and immunomodulatory efficacy of ginsenosides, including ginsenoside, derived from ginseng. (Balusamy et al., 2023). Although the safety and efficacy of the aforementioned plant-derived extracts have been established, their clinical application is limited by the suboptimal pharmacokinetic properties, including low solubility, inadequate membrane permeability, and metabolic lability. Improving the pharmacokinetic profile of the therapeutic agents by enhancing the solubility, providing protection, facilitating intracellular transport, and enabling sustained and controlled release is important for clinical application.

To enhance the drug efficacy and improve the bioavailability of active agents, nanotechnology-based formulations are extensively used to construct novel nanoagents (Mitchell et al., 2021; Nance et al., 2022). The encapsulation of herbal extracts within polymeric nanoparticles has been extensively validated to enhance the solubility, modulate drug release, reduce side effects, and enhance drug targeting and frequently results in increased therapeutic efficacy while shielding the active compounds from premature degradation (Kim et al., 2018; Chen X. et al., 2021; Wang et al., 2024). However, the application of encapsulation was commonly restricted from drug leakage and undesirable release performance. Hence, there is an urgent need for the development of a carrier that is relatively easy to prepare, has ideal release performance, and has excellent biocompatibility for delivery of plant-derived extracts. Among different nanocarriers, calcium carbonate nanoparticles (CaCO_3_ NPs) have gained much attention recently because of their excellent biocompatibility and biodegradability, easy preparation, and pH sensitivity (Qi et al., 2018). Many important studies have used CaCO_3_ NPs to deliver small-molecule drugs, genes, and proteins (Zhao et al., 2022; Kim et al., 2013). Thus, we hypothesized that CaCO_3_ NPs might be a suitable carrier for plant-derived extract delivery.

In this study, we use the extract of the root of ginseng, the fruit of *A. oxyphylla* Miq., and the stem of *M. speciosa* champ. as active agents to prepare a composite delivery system for achieving enhanced therapeutic efficacy. Glioblastoma multiforme is the most common malignant primary brain tumor with a median survival. We utilized CaCO_3_ NPs as carriers and optimized the synthesis of the extract-loaded composite nanoparticles for enhanced delivery performance in glioma treatment. The size and distribution, release curve, and therapeutic efficacy of ginseng CaCO_3_ NPs were verified, and they demonstrated an excellent anti-glioma effect. This is the first CaCO_3_ NP-based delivery strategy for naturally extracted ingredients of root, fruit, and stem, and it provides a promising strategy for glioma therapy using ginseng extract. We hope this study can provide inspiration for the development of novel delivery vehicles for plant extracts.

## 2 Materials and methods

### 2.1 Materials

Ginseng, *A. oxyphylla* Miq., and *M. speciosa* Champ. extracts were purchased from National Institutes for Food and Drug Control. 3-(4,5-Dimethylthiazol-2-yl)-2,5-diphenyltetrazolium bromide, dimethyl sulfoxide, cyclohexane, IGEPAL^®^ CO-520, calcium chloride, sodium carbonate, and chloroform were purchased from Sigma-Aldrich Chemical Co. (St. Louis, MO, United States). Cell culture plates and round coverslips were purchased from NEST Biotechnology Co., Ltd. (Wuxi, China). Fetal bovine serum (FBS) and high-glucose Dulbecco’s modified Eagle’s medium (DMEM) were obtained from Thermo Fisher Scientific (Chicago, IL, United States). C57BL/6 mice (5–6 weeks old, female) were purchased from Beijing Vital River Laboratory Animal Technology Company (Beijing, China). The experimental protocol was approved by the Committee on Ethical Animal Experiment at Hainan Medical University (HYLL-2023-379).

### 2.2 Preparation of CaCO_3_ NPs

The CaCO_3_ NPs loaded with ginseng, *A. oxyphylla* Miq., or *M. speciosa* Champ. were prepared by using a water in-oil reverse emulsion method (Qi et al., 2018; Li et al., 2010). In brief, 500 μL of 300 mM CaCl_2_ and the aqueous extracts of ginseng, *A. oxyphylla* Miq., or *Milletia speciosa* Champ. were dispersed in 50 mL of the cyclohexane/IGEPAL CO-520 (70/30, V/V) “solution to form a water-in-oil reverse microemulsion. The “carbonate microemulsion” was prepared by combining 500 μL of 300 mM Na_2_CO_3_ in another 50-mL oil phase. Two microemulsions were combined and mixed overnight at room temperature. Then, 100 mL of absolute ethanol was added to the microemulsion and centrifuged at 10,000 g for 40 min to pellet to remove the cyclohexane and surfactant. The drug-loaded CaCO_3_ NPs werewashed 2–3 times using ethanol and suspended in water for storage.

### 2.3 Characterization of CaCO_3_ NPs

The hydrodynamic sizes of CaCO_3_ NPs were measured via dynamic light scattering (Zetasizer Nano ZS, Malvern Instruments Ltd., UK). The morphologies of NPs were observed via transmission electron microscopy (TEM, JEOL 100CX II, Japan). The extent of entrapment efficiency was quantified using a spectrophotometer (UV-2600, Shimzadu, Kyoto, Japan). The absorbance values of ginseng*, A. oxyphylla* Miq., and *M. speciosa* Champ. aqueous extracts were measured at 540 nm, 485 nm, 256 nm, respectively. The entrapment efficiency was calculated based on the following equation:

$$\%\text{ Entrapment Efficiency}=\left(\text{Drug loaded}/\text{Drug added}\right)\times 100.$$

The pH-dependent release of ginseng*, A. oxyphylla* Miq., or *M. speciosa* Champ from CaCO_3_ NPs was studied using a dialysis method at 37°C. Phosphate-buffered saline (PBS) solutions with pH 7.4 and pH 5.5 were used as the media for simulating normal blood/tissue and tumoral lysosomal acidic conditions. CaCO_3_ NPs loaded with ginseng*, A. oxyphylla* Miq., or *M. speciosa* Champ. were placed into pretreated dialysis bags (MW cutoff of 14 kDa). The dialysis bags were placed into brown bottles containing 100 mL of PBS solutions of different pH values. These bottles were shaken at 37°C while being shielded from light. Samples were withdrawn at various intervals and replaced with an equal volume of fresh buffer. The amount of the released drug was analyzed using a spectrophotometer.

### 2.4 *In vitro* cytotoxicity

Cell viability was determined via MTT assay. GL261 cells were seeded in 96-well plates at 2 × 10^4^ cells per well for overnight culture. After 24 h of treatment with the drugs (ginseng aqueous extracts, *A. oxyphylla* Miq. aqueous extracts, *M. speciosa* Champ. aqueous extracts, ginseng CaCO_3_ NPs, *A. oxyphylla* Miq. CaCO_3_ NPs, and *M. speciosa* Champ. CaCO_3_ NPs). The MTT solution was added. After an additional 4 h of incubation, the supernatants were removed carefully, and 150 μL of DMSO was added to each well. The absorbance value was measured at 570 nm using the SpectraMax M5 microplate reader.

For apoptosis analysis via flow cytometry, GL261 cells (5 × 10^5^ per well) were seeded on six-well plates. Then, cells were treated with CaCO_3_ NPs for 12 h. The cells were harvested by trypsinization and were stained using the Annexin V-FITC Apoptosis Detection Kit (KeyGEN Biotech, Nanjing, China) based on the manufacturer’s protocol. Stained cells were immediately analyzed on a BD Accuri C6 Flow Cytometer.

### 2.5 *In vivo* antitumor efficacy

GL261 cells were transformed using the luciferase gene (GL261-Luc) for constructing the glioma mouse model. To construct the GL261 intracranial orthotopic glioblastoma mouse model, C57BL/6 mice were anesthetized with isoflurane and placed in a stereotactic instrument. Then, 1.0 × 10^5^ GL261 cells in a 5 µL volume were injected into the right striatum (1 mm anterior, 2 mm right lateral from bregma, and 3.5 mm deep). The skin was sealed using surgical glue. Real-time bioluminescence imaging was used to evaluate the therapeutic efficiency. The GL261-Luc mice were randomly divided into three groups (n = 5 per group), which were intravenously injected with three injections of PBS, ginseng aqueous extract, and ginseng CaCO_3_ NPs on days 5, 8, and 11 after implantation, containing ginseng (5 mg kg^−1^) per dose. The bioluminescence signal was used to evaluate the therapeutic efficiency at fifth, 10th, and 15th days after tumor implantation through the IVIS Spectrum system. Throughout the study, mice were weighted regularly. For safety detection, 500 μL of blood was collected from the tail vein of each mouse, and the blood serum was isolated to analyze some biochemical indicators containing aspartate transaminase (ALT), aspartate aminotransferase (AST), blood urea nitrogen (BUN), and creatinine (CREA). Major organs were collected and processed for immunohistochemical analysis.

### 2.6 Statistical analysis

All data were collected from three independent experiments and then expressed as means ± standard deviation (SD). Statistical significance was analyzed using an unpaired, two-tailed Student’s t-test, as well as one-way or two-way ANOVA analysis using GraphPad Prism 5.0 (San Diego, CA, United States). Statistical significance thresholds were set at *p < 0.05, **p < 0.01, and ***p < 0.001.

## 3 Results and discussion

### 3.1 Preparation and characterization of CaCO_3_ NPs

**FIGURE 1:**
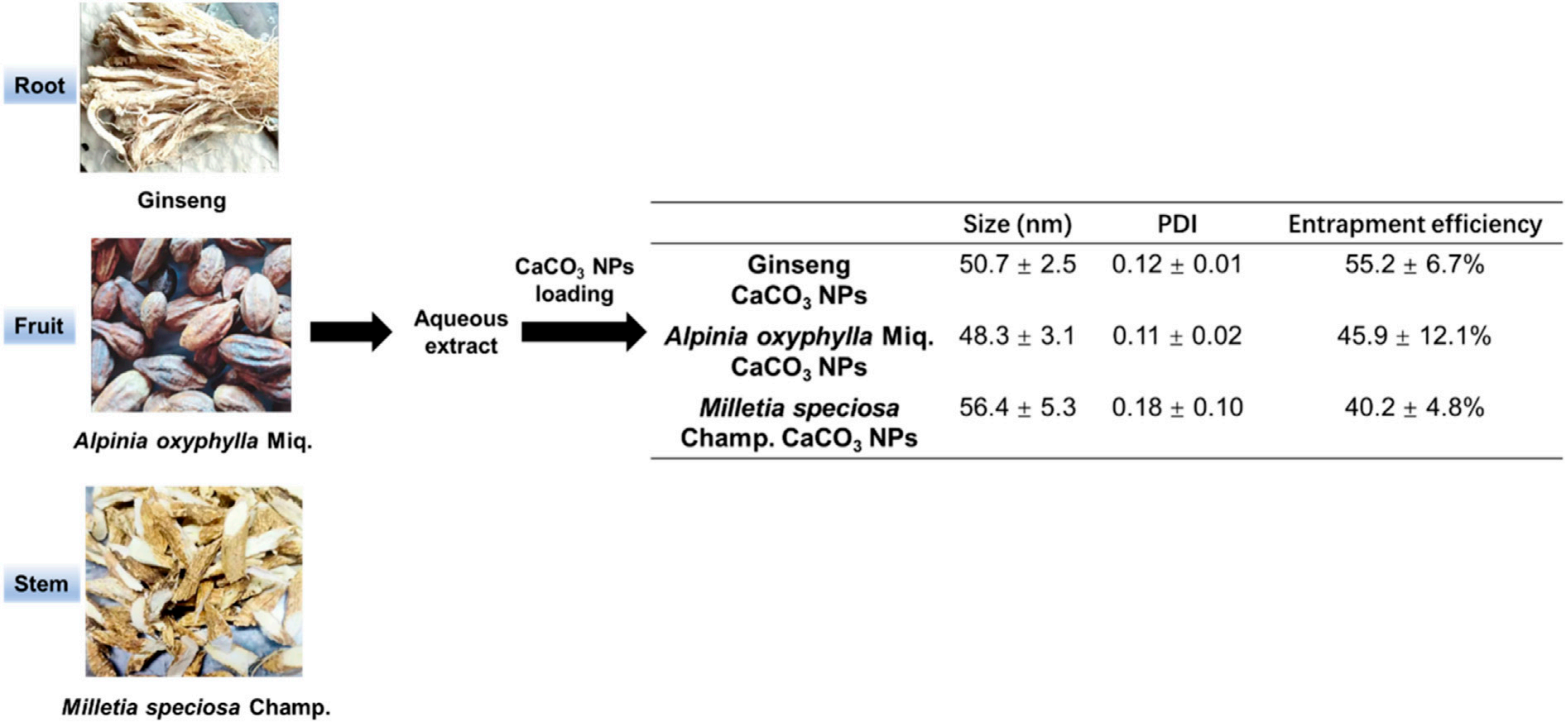
Schematic illustration of the construction of CaCO_3_-based composite nanoparticles. The aqueous extracts derived from the root of the ginseng, the fruit of *A. oxyphylla* Miq., and the stem of *M. speciosa* champ. were prepared as loaded drugs for the construction of composite nanoparticles. The size, polydispersity (PDI), and entrapment efficiency (EE) of the corresponding composite nanoparticles (ginseng CaCO_3_ nanoparticles, *A. oxyphylla* Miq. CaCO_3_ nanoparticles, and *M. speci*osa champ. CaCO_3_ nanoparticles) were measured for the characterization of the load performance. Data are expressed as mean ± SD (n = 5).

To construct the composite nanoparticles, we chose the aqueous extracts derived from the root of the ginseng, the fruit of *A. oxyphylla* Miq., and the stem of *M. speciosa* champ. as the active agents. CaCO_3_ NPs loaded with the aqueous extracts were prepared through a reverse microemulsion method (Figure 1). The particle size and polydispersity indexes (PDI) of three kinds of CaCO_3_ NPs were determined via dynamic light scattering (DLS). As shown in Figure 1, the results showed that the average particle sizes of ginseng, *A. oxyphylla* Miq., and *M. speciosa* Champ. CaCO_3_ NPs were 50.7 ± 2.5 nm, 48.3 ± 3.1 nm, and 56.4 ± 5.3 nm, respectively. The PDI measured in PBS were 0.12 ± 0.01, 0.11 ± 0.02, and 0.18 ± 0.10, respectively, indicating a uniform particle size and ideal distribution in the physiological environment. We next measured the loading efficacy of the composite nanoparticles. The entrapment efficiencies (EE) of ginseng, *A. oxyphylla* Miq., and *M. speciosa* Champ. were measured as 55.2% ± 6.7%, 45.9% ± 12.1%, and 40.2% ± 4.8%, respectively, implying that the extract of ginseng had relatively higher EE compared with the other two kinds of extracts. By optimizing the preparation conditions, we achieved high loading efficiency, providing assurance for subsequent applications.

**FIGURE 2:**
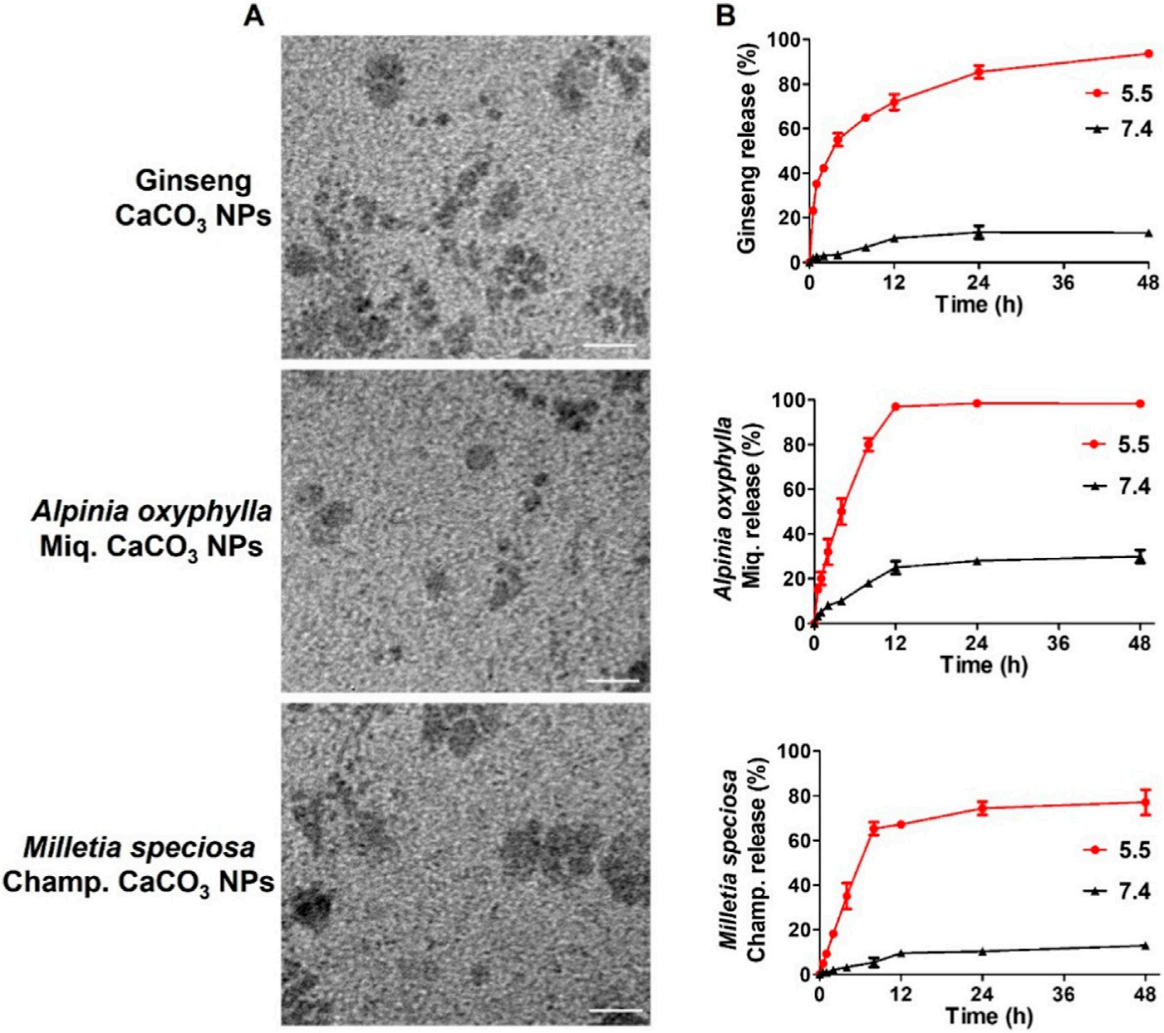
Preparation and characterization of CaCO_3_-based composite nanoparticles. **(A)** TEM image of CaCO_3_ NPs. Scale bar, 100 nm. **(B)** Cumulative drug release from CaCO_3_ NPs.

**FIGURE 3:**
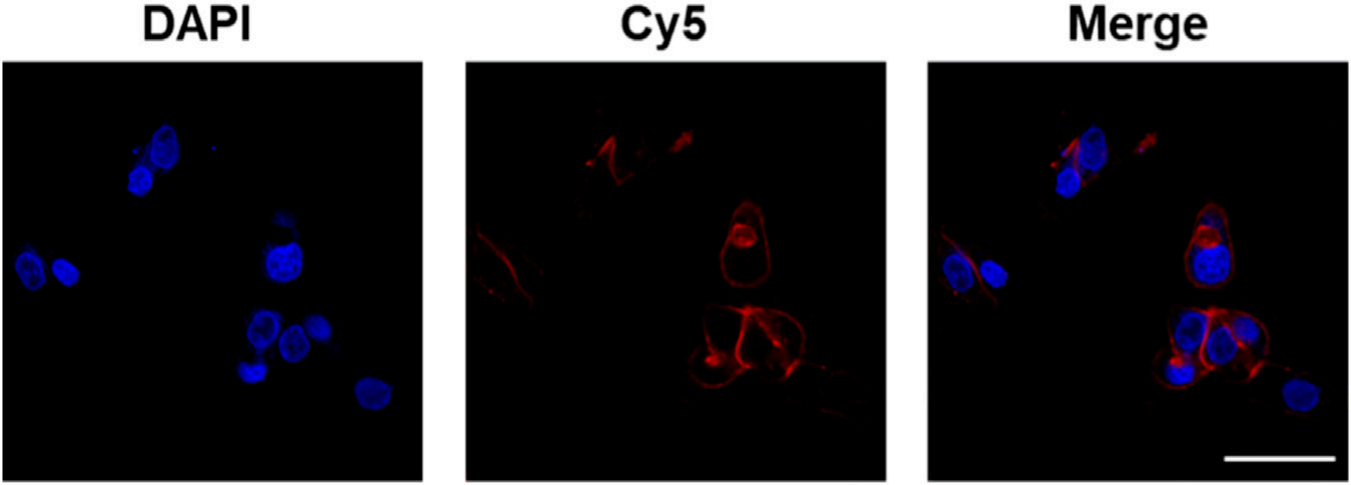
Confocal fluorescence images of GL261 cells after incubation with Cy5-loaded CaCO_3_ NPs. Scale bar: 50 µm.

We next characterize the morphology and release performance of the nanoparticles. The stability of the nanoparticles was demonstrated to be good, allowing for stable storage at room temperature over a 2-week period. The results of transmission electron microscopy (TEM) revealed that ginseng, *A. oxyphylla* Miq., and *M. speciosa* Champ. CaCO_3_ NPs were spherical in shape, with a relatively small size of approximately 30 nm, as shown in Figure 2A and Supplementary Figure S1. The relatively larger sizes measured via DLS than those via TEM may be attributed to the surface hydration of NPs. In the release performance test, the pH-sensitive release of ginseng, *A. oxyphylla* Miq., and *M. speciosa* Champ. from CaCO_3_ NPs was analyzed via dialysis. Phosphate-buffered saline (PBS) solutions at pH 7.4 and 5.5 were utilized to replicate the physiological conditions of normal healthy tissue and the acidic environment of tumor tissues, respectively. After 48 h, 93.7% of ginseng, 98.3% of *A. oxyphylla* Miq., and 77.2% of *M. speciosa* Champ. were released from the corresponding CaCO_3_ NPs at pH 5.5. In contrast, only 13.3% of ginseng, 30.0% of *A. oxyphylla* Miq., and 12.9% of *M. speciosa* Champ. were released at pH 7.4 (Figure 2B). These data revealed that low pH (5.5) triggered drug release from CaCO_3_ NPs. Moreover, ginseng CaCO_3_ NPs have a better controlled-release performance. The stability of *M. speciosa* Champ. CaCO_3_ NPs was assessed in physiological environments and PBS. As shown in Figure SX, there was no significant change in size after 7 days (Supplementary Figure S2). We next used Cy5-loaded CaCO3 NPs to simulate drug internalization. As shown in Figure 3, the effectiveness of internalization was tested in GL261 cells. Through the systematic analysis of the abovementioned experimental results, we found that the size distribution and release properties of ginseng particles make them most suitable as a therapeutic carrier among the three types of particles.

### 3.2 *In vitro* cytotoxicity of ginseng CaCO_3_ NPs

**FIGURE 4:**
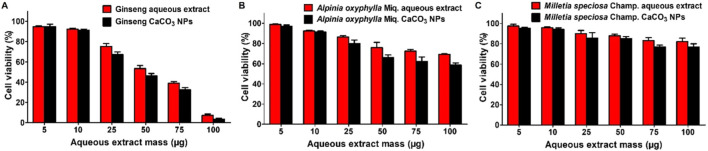
Viabilities of GL261 cells after different treatments for 24 h. **(A)** Cell viability of ginseng aqueous extracts and ginseng CaCO_3_ NPs. **(B)** Cell viability of *A. oxyphylla* Miq. aqueous extracts and *A. oxyphylla* Miq. CaCO_3_ NPs. **(C)** Cell viability of *M. speciosa* Champ. aqueous extracts and *M. speciosa* Champ. CaCO_3_ NPs.

Next, the cytotoxicities of ginseng*, A. oxyphylla* Miq., and *M. speciosa* Champ. aqueous extracts and ginseng, *A. oxyphylla* Miq., and *M. speciosa* Champ. CaCO_3_ NPs were evaluated in GL-261 cells via MTT assay. As can be seen in Figure 4, the treatment of the ginseng aqueous extract exerted greater inhibition of cell viability compared with *A. oxyphylla* Miq. or *M. speciosa* Champ. in GL-261 cells. At 50 μg of ginseng, only 53.5% and 46.4% cells survived with the treatment of ginseng aqueous extract and ginseng CaCO_3_ NPs, respectively. In contrast, *A. oxyphylla* Miq. and *M. speciosa* Champ. only had mild toxicity. Furthermore, the loading of drugs into CaCO_3_ NPs could partly increase the toxicity. Thus, ginseng CaCO_3_ NPs were chosen for further evaluation.

**FIGURE 5:**
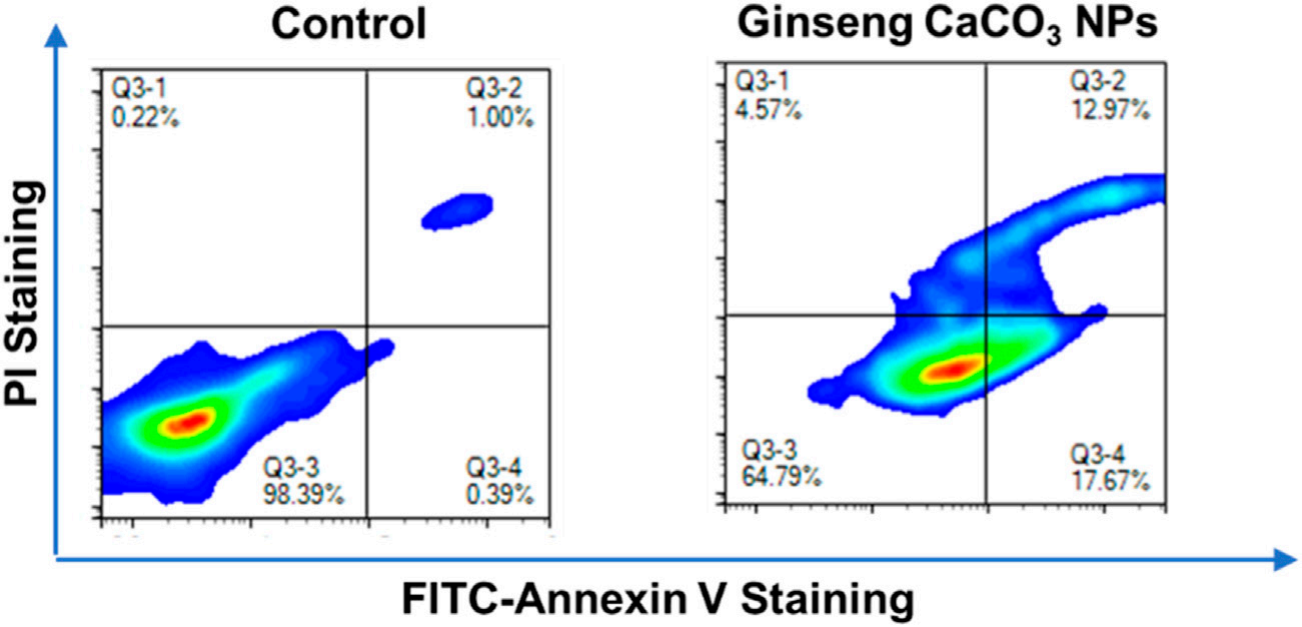
Cell apoptosis analysis via annexin V-FITC/PI double staining.

To further quantify cell apoptosis caused by the treatment with ginseng CaCO_3_ NPs against GL261 cells, flow cytometry analysis was utilized. After incubation with ginseng CaCO_3_ NPs for 12 h, GL261 cells were stained with annexin V and propidium iodide (PI). As shown in Figure 5, ginseng CaCO_3_ NPs induced up to 30.6% overall apoptosis, further proving the cytotoxicity in GL261 cells.

### 3.3 Tumor inhibitory effects *in vivo*

**FIGURE 6:**
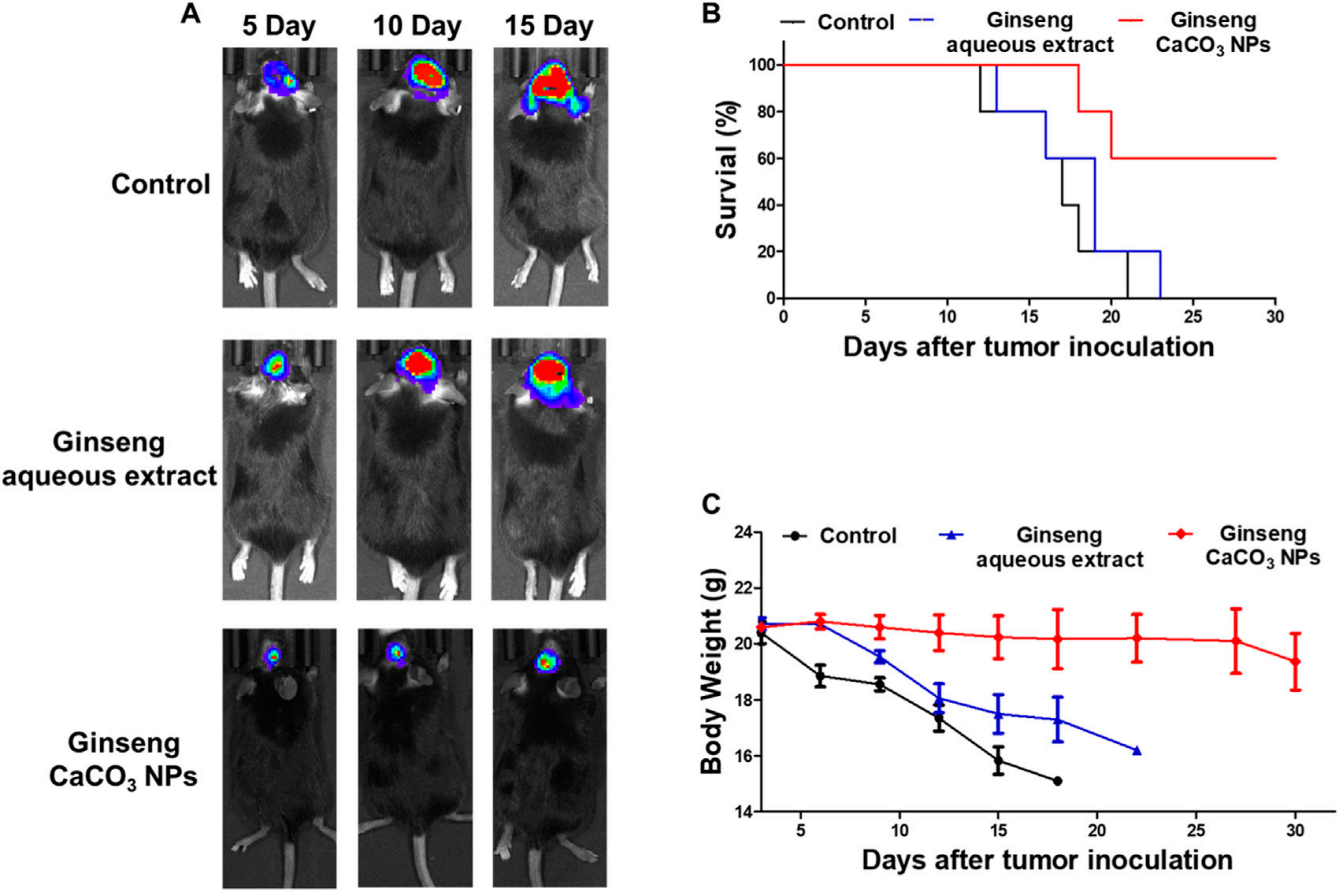
*In vivo* anti-glioma activity of ginseng CaCO_3_ NPs. **(A)** Representative bioluminescence images of GL261-Luc glioma-bearing mice treated via different groups. **(B)** Survival curve for the mice (n = 5 mice per group). **(C)** Body weight change in different groups.

**FIGURE 7:**
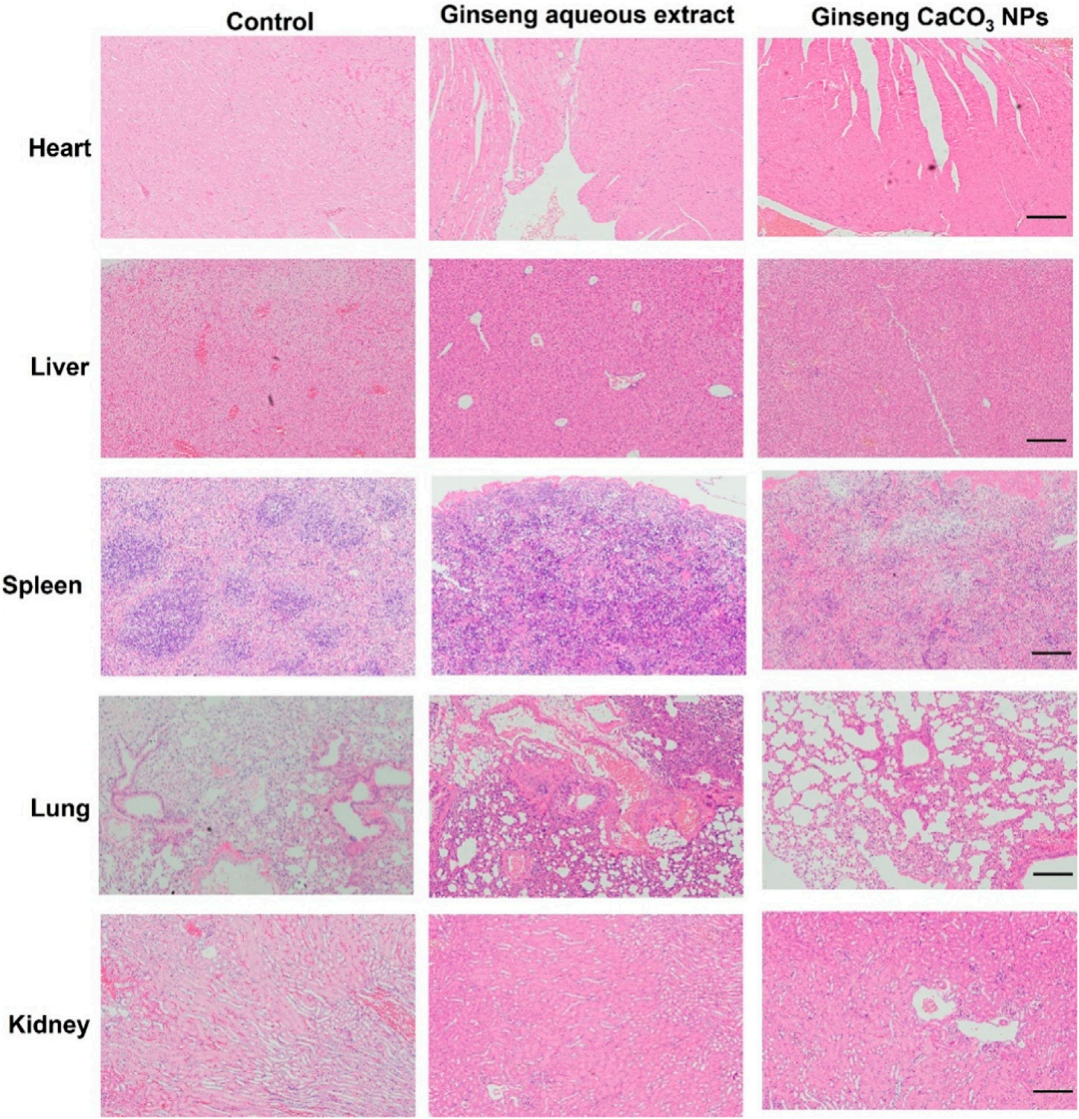
H&E staining of major organs after treatments. Scale bar: 200 µm.

Encouraged by the antitumor effect *in vitro* of ginseng CaCO_3_ NPs, we next investigated the antitumor efficacy *in vivo* through an orthotopic GL261-Luc glioma mouse model. As shown in Figure 6A, IVIS Spectrum demonstrated rapid tumor growth in the PBS or ginseng aqueous extract-treated group, whereas the bioluminescence signals of the ginseng CaCO_3_ NP group were obviously weaker than those of the PBS or ginseng aqueous extract-treated groups, indicating ginseng CaCO_3_ NPs hold the strongest antitumor effect. Moreover, the survival study also proved that ginseng CaCO_3_ NPs could prolong mice survival and lead to a 60% survival rate in 30 days (Figure 6B). The body weight of mice was deeply affected by different treatments, which is similar to the trend of survival rate (Figure 6C). No significant alterations were observed in the blood biochemical markers of liver and kidney toxicity, including alanine aminotransferase (ALT), aspartate aminotransferase (AST), blood urea nitrogen (BUN), and creatinine (CREA), in all therapy groups, indicating that no significant renal and liver toxicity was observed after ginseng CaCO_3_ NP treatment (Supplementary Figure S3). Furthermore, no significant histopathological changes were observed in the harvested heart, liver, spleen, lung, and kidney via HE staining (Figure 7). All these results proved that ginseng CaCO_3_ NPs could be a safe and effective strategy for glioma treatment.

## 4 Conclusion

We used extracts from the roots of ginseng, the fruits of *A. oxyphylla* Miq., and the stems of *M. speciosa* Champ. as bioactive constituents to fabricate a composite delivery system that aims to improve the therapeutic efficacy. We explored a novel approach using CaCO_3_ NPs to combat the defects in the development of the plant extract-involved drug delivery carriers. The size, distribution, release kinetics, and therapeutic efficacy of the composite nanoparticles were characterized, and ginseng CaCO_3_ NPs exhibited great potential in glioma therapy. This study represents the first to use a CaCO_3_ NP-based delivery strategy for the natural extracts of roots, fruits, and stems, offering a promising therapeutic approach for glioma treatment using ginseng extract. We believe that the development of novel nanoformulations can promote the application of natural products in disease treatment.

## Data Availability

The original contributions presented in the study are included in the article/Supplementary Material; further inquiries can be directed to the corresponding authors.
