# Supplementary material for: Composite nanoparticle-based vesicles achieve enhanced delivery effects of the natural plant extract of the root, stem, and fruit
Source: Front Chem. 2025 Mar 17;13:1552298. doi: 10.3389/fchem.2025.1552298 (PMC11955615; doi:10.3389/fchem.2025.1552298)
Supplement: Supplementary file 1 [file DataSheet1.docx]

Supplementary Material

Composite Nanoparticles-based Vesicles Achieve Enhanced Delivery Effect of the Natural Plant Extract of Root, Stem, and Fruit

**Xiaodong Zhuang^1^, Ting Ma^1^, Risheng Liu^1^, Pengxuan Zhao^2*^. Xingyue Fang^2*^, Liangjiu Huang^1*^**

^1^ Department of Clinical Pharmacy, Hainan Cancer Hospital, Haikou, 570312, China

^2^ Department of Pharmacy & Engineering Research Center of Tropical Medicine Innovation and Transformation, The First Affiliated Hospital of Hainan Medical University, Haikou, 570102, China

*** Correspondence:**Liangjiu Huang

hyfzl00603@muhn.edu.cn

# Supplementary Figures


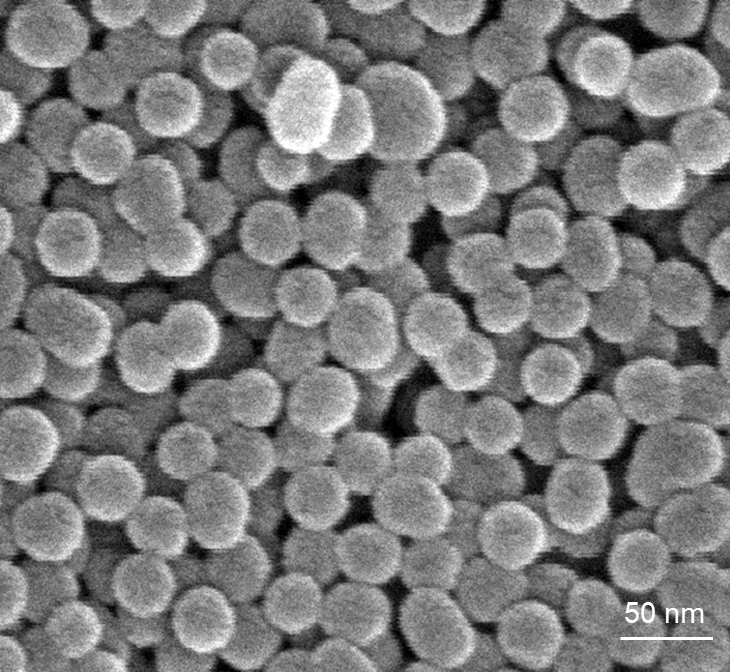


**Supplementary Figure S1**. SEM image of *Milletia speciosa* Champ. CaCO_3_ NPs.

**Supplementary Figure S2.** Time-dependent stability of *Milletia speciosa* Champ. CaCO_3_ NPs in DMEM/FBS and PBS at 37 °C.


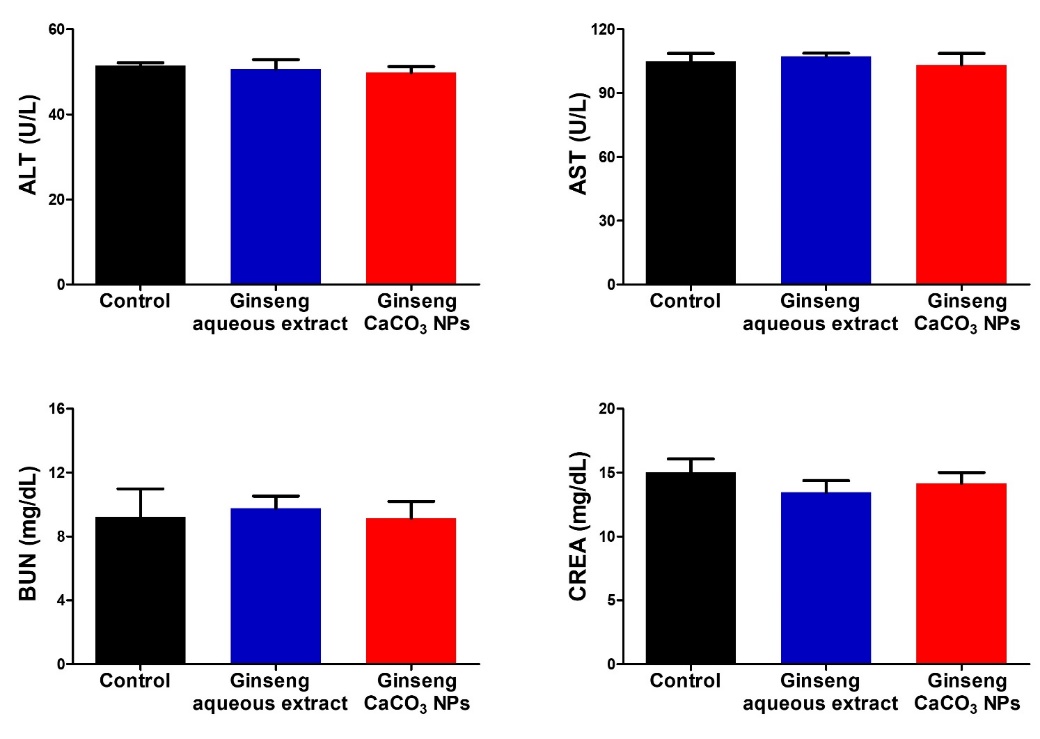


**Supplementary Figure 3**. Effect of different treatments on serum ALT, ALS, BUN, and CREA levels.
